# Dual Functions of the C5a Receptor as a Connector for the K562 Erythroblast-Like Cell-THP-1 Macrophage-Like Cell Island and as a Sensor for the Differentiation of the K562 Erythroblast-Like Cell during Haemin-Induced Erythropoiesis

**DOI:** 10.1155/2012/187080

**Published:** 2012-12-30

**Authors:** Hiroshi Nishiura, Rui Zhao, Tetsuro Yamamoto

**Affiliations:** Department of Molecular Pathology, Faculty of Life Science, Kumamoto University Graduate School, Honjyo 1-1-1, Chuou-ku, Kumamoto 860-8556, Japan

## Abstract

The transcriptional nuclear factor binding to the Y box of human leukocyte antigen genes (NF-Y) for the *C5a receptor* (*C5aR*) gene is active in erythroblasts. However, the roles of the *C5aR* in erythropoiesis are unclear. We have previously demonstrated that apoptotic cell-derived ribosomal protein *S19* (*RP S19*) oligomers exhibit extraribosomal functions in promoting monocyte chemotaxis and proapoptosis via the *C5aR* without receptor internalisation. In contrast to the extraribosomal functions of the *RP S19*, a proapoptotic signal in pro-EBs, which is caused by mutations in the *RP S19* gene, is associated with the inherited erythroblastopenia, Diamond-Blackfan anaemia. In this study, we detected *C5aR* expression and *RP S19* oligomer generation in human erythroleukemia K562 cells during haemin-induced erythropoiesis. Under monocell culture conditions, the differentiation into K562 erythrocyte-like cells was enhanced following the overexpression of Wild-type *RP S19*. Conversely, the differentiation was repressed following the overexpression of mutant *RP S19*. An *RP S19* oligomer inhibitor and a *C5aR* inhibitor blocked the association of the K562 basophilic EB-like cells and the THP-1 macrophage-like cells under coculture conditions. When bound to *RP S19* oligomers, the *C5aR* may exhibit dual functions as a connector for the EB-macrophage island and as a sensor for EB differentiation in the bone marrow.

## 1. Introduction

The homeostasis of haematopoietic stem cells is partially maintained by the differentiation stage-specific activation of transcription factors [1]. In human leukocyte antigen genes, the binding of NF-Y to the CCAAT motif upstream of the promoter at the terminal differentiation stage of myeloid cells is crucial for the expression of the C5aR protein, a member of the Gi protein-coupled receptor (GiPCR) family [2]. In leukocytes, when bound to complement C5-derived anaphylatoxin C5a at the acute inflammatory phase, the C5aR functions as a proinflammatory factor and exhibits receptor internalisation [3]. The G*βγ* subsets transmit an extracellular signal-regulated kinase 1/2 (ERK1/2) signal via phospholipase C. Therefore, the activation of the C5aR is limited by the binding of arrestin to its C-terminal intracellular regions, which are phosphorylated sequentially by protein kinase C and G protein-coupled receptor kinase 2, downstream of ERK1/2 [4]. Recently, the neutrophil C5aR was shown to function briefly as an antiapoptotic factor that phosphorylates the pro-apoptotic Bax on the mitochondrial membrane, inducing the translocation of Bax to the 14-3-3 protein for degradation by the 26S proteasome [5]. Therefore, it has been suggested that C5a attracts neutrophils via the neutrophil C5aR, and the antiapoptotic signal is briefly transmitted in neutrophils to prolong of the lifespan of the cell.

We have previously demonstrated that NF-Y can be activated in any apoptotic cell and that RP S19 is cross-linked at Lys122 and Gln137 by the activated type II tissue transglutaminase (TGII) [6]. The activation site in the C5aR bound to the Leu_131_ AspArg moiety of RP S19 oligomers functions as a pro-apoptotic factor for apoptotic cells and as a chemotactic factor for monocytes/macrophages in the absence of receptor internalisation. The G*βγ* subsets of the monocyte C5aR transmit the p38 mitogen-activated protein kinase (p38MAPK) signals, indicating that the C5aR C-terminus is not phosphorylated. When RP S19 oligomers bind to the C5aR on apoptotic cells (including neutrophils), the additional binding of the RP S19 C-terminus to an inhibitory molecule inhibits p38MAPK signalling. However, the *de novo* synthesis of the regulator of G protein signalling 3 (RGS3) is initiated to inhibit the microenvironment factor-dependent ERK1/2 signalling mediated by the constitutively activated GPCRs. Therefore, we suggest that the RP S19 oligomers released from apoptotic cells attract macrophages for the connection between the *de novo* synthesised C5aR on apoptotic cells and the monocyte C5aR on macrophages without receptor internalisation. The pro-apoptotic signal is transmitted continuously in apoptotic cells for the execution of apoptosis. 

Erythropoiesis is maintained primarily by transcription factors via the differentiation stage-specific activation of growth factor receptors [7]. Early erythroid progenitors (burst-forming unit-erythroid, BFU-E) are sensitive to GPCR 48 or the receptor-type tyrosine kinases C-kit and FLT3, which function as transcription factors for the *antiapoptotic protein* genes [8, 9]. In contrast, late erythroid progenitors (colony-forming unit-erythroid, CFU-E) and morphological erythroid precursors (proerythroblast, pro-EB) are sensitive to the activities of the Fas ligand receptor, which functions as an activator of pro-apoptotic caspase-3 [10–12]. Moreover, monocyte chemoattractant protein-2/4, released from the CFU-E-derived EBs, was recently suggested to contribute partially to erythropoiesis through the formation of the EB-macrophage islands [13]. However, an inherited erythroblastopenia in a case of Diamond-Blackfan anaemia was recently reported to be associated with mutations in at least *8 different ribosomal protein* genes [14]. The number of peripheral blood erythrocytes in FVB/N mice is decreased by the dominant negative effect of overexpressing the Arg62Trp mutant RP S19. These data confirm a role for the constitutive pro-apoptotic signal through a defect in the ribosome formation mediated by the mutant RP S19 at the BFU-E stage [15]. However, the roles of the differentiation stage-specific activation of pro-apoptotic signals and the formation of the EB-macrophage islands under normal conditions are not clearly understood.

A number of interesting studies report the erythroid-specific transcriptional activation of the *growth factor-independence-1B* and the *nucleolar spindle-associated protein* genes that contribute to the functional cooperation between GATA-1 and NF-Y in immature human erythroleukemia K562 cells and mature erythroleukemia MEL cells [16, 17]. If the C5aR is expressed during erythropoiesis, the RP S19 oligomer-induced extraribosomal functions will regulate the intracellular pro-apoptotic signal via the C5aR on EBs and the interaction of basophilic-EBs with the macrophages for a long period in the absence of receptor internalisation, as shown previously in apoptotic cells. 

In this study, to confirm the similarity between the morphological changes in K562 cells after the induction of apoptosis using MnCl_2_ and the differentiation using iron-containing porphyrin (haemin), we first transfected the K562 cells with the enhanced-green-fluorescent-protein- (EGFP-) fused ANXA3 cDNA as a marker for apoptosis [18, 19]. The haemin-induced differentiation of the K562 cells was limited to the basophilic-EB-like cell stage by the constitutively active ERK1/2 signalling downstream of a *Bcr-Abl* chimeric gene. Therefore, a blockade of the constitutively activated ERK1/2 signalling pathway would allow further cell differentiation. Next, we investigated the effects of RP S19 and RGS3 overexpression under monocell culture conditions and the EB-macrophage islands under coculture conditions on overcoming the haemin-induced differentiation defect in the K562 cells. 

## 2. Materials and Methods

### 2.1. Antibodies and Recombinant C5a

The phosphorylated and unphosphorylated anti-ERK1/2 and anti-p38MAPK rabbit IgGs were from Cell Signaling Technology (Boston, USA). The anti-ventricular myosin light chain 3 (LC3) rabbit IgG was from Sigma (Tokyo, Japan). The anti-C5aR, anti-TGII, anti-RGS3 and anti-actin rabbit IgGs, the fluorescein isothiocyanate- (FITC-) conjugated anti-C5aR mouse IgG, and the FITC- and HRP-conjugated anti-rabbit IgG goat IgGs were from Santa Cruz Biotechnology (CA, USA). The FITC-conjugated anti-transferrin receptor (CD71) mouse IgG and the phycoerythrin- (PE-) conjugated anti-glycophorin A (CD235a) mouse IgG were from BD (Tokyo, Japan). 

The recombinant C5a was prepared using the pET32a-Rosetta gami(B) Lys-S system to generate anti-C5a rabbit IgGs [20]. Briefly, human C27R C5a cDNA, prepared using the polymerase chain reaction, was inserted into the pET32a vector between the BamH1 and EcoR1 sites. The expression host, *E. coli* Rosetta-gami(B) Lys-S cells transformed with pET32a-C27R-C5a cDNA, was cultured for 6 hr and reincubated for another 5 hr in the presence of 1 mM isopropyl 1-thio-*β*-D-galactoside. The Trx-His-S-Tag C27R-C5a protein recovered from the cultured *E. coli* cells was purified using the Hi-Trap chelating HP column (GE, Tokyo, Japan) preloaded with 1 mL 100 mM NiSO_4_. The S-Tag C27R-C5a protein dialysed against thrombin was purified using the Hi-Trap benzamidine FF column and the Hi-Trap heparin HP column (GE). The purity of the protein was confirmed by sodium dodecyl sulphate polyacrylamide gel electrophoresis (SDS-PAGE) and western blotting using anti-RP S19 rabbit IgG. The anti-C5a rabbit IgG was raised in a male New Zealand White rabbit (Kyudo, Kumamoto, Japan). The blood was collected for 2 months after injection of 500 *μ*L of 1 mg/mL Trx-His-S-Tag C27R-C5a protein in PBS mixed with 500 *μ*L of Freund's complete or incomplete adjuvant (Sigma), and the serum was prepared using standard protocols. The IgG was separated from the sera using a Hi-Trap protein-G HP column (GE) and was stored at a concentration of 4 mg/mL in PBS at −80°C.

### 2.2. Cells

The human leukaemia K562 and monocytic THP-1 cells were obtained from the RIKEN BioResource Center in Japan. The K562 cells were differentiated into K562 basophilic-EB-like cells using 30 *μ*M haemin, and the monocytic THP-1 cells were differentiated into THP-1 macrophage-like cells using 160 nM phorbol-12-myristate-13-acetate (PMA) (Sigma) [21]. To select for apoptosis-resistant clones (EGFP-ANXA3 K562^R^ cells) using limiting dilution methods, the EGFP-ANXA3 K562 cells were cultured for 3 days in the presence of 1 mM MnCl_2_ [18, 22]. To examine the haemin-induced differentiation of the K562 cells using fluorescence-activated cell sorting (FACS), the cells were stained with FITC-conjugated anti-CD71 and PE-conjugated anti-CD235a mouse IgGs for 30 min at 4°C.

### 2.3. Vectors and K562 Transformations

The ANXA3 cDNA, prepared from human hepatoma HepG2 cells using RT-PCR, was inserted into the pENTR4-H1 vector. The H1-ANXA3 cassette was placed within the RfA region of the pCS-RfA-EG vector by LR recombination (Life, Tokyo, Japan). The 293T cells (5 × 10^6^ cells/mL) were maintained in DMEM medium containing 10% FBS in poly-lysine-coated 10-cm tissue culture plates for 24 hr before adding fresh medium. To produce the HIV-1-based lentivirus packaged with the CSIIE-ANXA3 cDNA, a plasmid DNA solution (450 *μ*L) containing 17 *μ*g of the packaging plasmid and 10 *μ*g of the VSV-G and Rev-expression plasmids was mixed with 50 *μ*L 2.5 M CaCl_2_ and 500 *μ*L 2x HEBS (1.5 mM Na_2_HPO_4_, 280 mM NaCl, and 50 mM HEPES) and was used to transfect the 293T cells. The HIV-1-based lentivirus was recovered by ultracentrifugation and resuspended in 50 *μ*L of HBSS medium. To establish the stably transformed K562 cells harbouring the CSIIE-ANXA3 cDNA, we infected 100 *μ*L of the cells (1 × 10^6^ cells/mL) with the lentivirus at a multiplicity rate of infection of 10, and the stably transfected clones were screened using 1 mM Zeocin.

We constructed the pCAGGS-IRES neomycin-resistant vectors containing the Wild-type RP S19, Gln137Asn mutant RP S19, and RGS3 cDNAs [23]. In addition, the pDsRed vector was used to label the nucleus (Takara, Kyoto, Japan). To establish the transformed K562 cells, the cells were suspended in DMEM medium at a density of 5 × 10^6^ cells/mL and were electroporated with 40 *μ*g of linearised plasmid DNA using a Gene Pulsar II electroporator (Bio-Rad, CA, USA) in a 4 mm gap cuvette at 200 V and 950 *μ*F. Of the 24 positive clones that were screened using 250 *μ*M Geneticin, the best clone was selected based on the cell growth rate (data not shown). 

### 2.4. SDS-PAGE and Western Blotting

The ectosome fractionation was performed in accordance with the isolation protocol described previously [24]. After precipitation with cold acetone, the samples were resolved by SDS-PAGE and were silver-stained. Following the SDS-PAGE, the K562 proteins were electrophoretically transferred from the gel onto an Immobilon Transfer Membrane (Millipore, MA, USA) using a Semi-Dry Electroblotter (Sartorius, Göttingen, Germany) at 15 V for 90 min. The membrane was treated with 4% Block Ace (Dainippon, Osaka, Japan) for 30 min at 22°C. The first immunoblotting reaction was performed using rabbit IgG antibodies (200 ng/mL) in PBS containing 0.03% Tween-20 for 1 hr at 22°C. After washing, the second immunoblotting reaction was performed using the HRP-conjugated goat anti-rabbit IgGs (20 ng/mL) for 30 min at 22°C. The enhanced chemiluminescence reaction was performed using the ECL Plus Western Blotting Detection System (GE).

### 2.5. Statistical Analysis

The results from representative experiments were confirmed by performing multiple experiments using a minimum of triplicate samples. The statistical significance was calculated using the nonparametric or parametric tests with two-way analysis of variance. The values were expressed as the means ± SD. A *P* value of <0.05 was considered statistically significant and is shown as *P < 0.05; a *P* value of <0.01 is labelled **P < 0.01. 

## 3. Results

### 3.1. Common Morphological Changes in K562 Cells during MnCl_2_-Induced Apoptosis and Haemin-Induced Erythroid Differentiation

**Figure 1:**
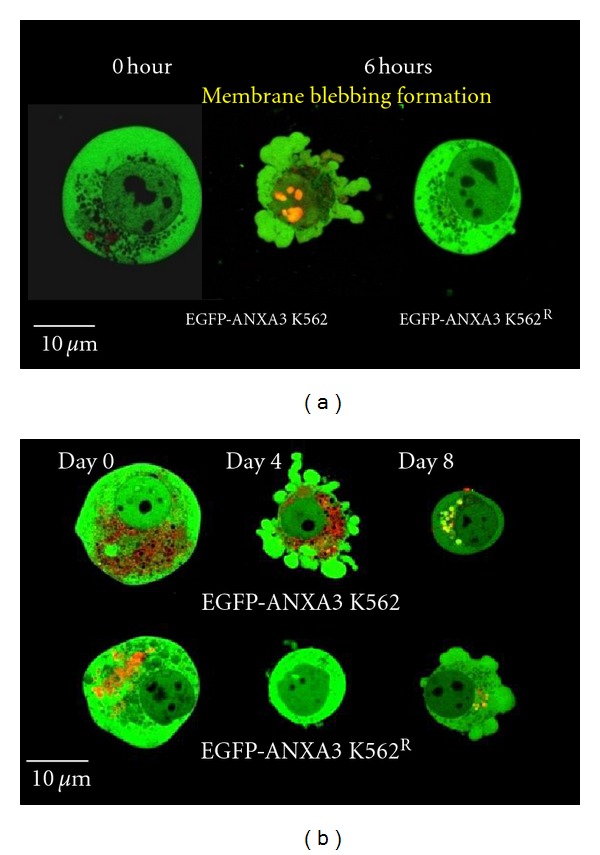
Common morphology between K562 cells after MnCl_2_ and haemin treatments. ((a) and (b)) K562 and K562^R^ cells expressing EGFP-ANXA3 were observed under CLSM at several time points after MnCl_2_ or haemin induction (*n* = 6).

To demonstrate the morphological changes that occur during the MnCl_2_-induced apoptosis and haemin-induced differentiation of the K562 cells, we prepared EGFP-ANXA3 K562 cells and EGFP-ANXA3 K562^R^ cells. The markers characteristics of programmed cell death, cell shrinkage, and membrane blebbing were detected in the EGFP-ANXA3 K562 cells using confocal laser microscopy (CLSM) (FLUOVIEW FV300, Olympus, Tokyo, Japan) at 6 hr after MnCl_2_ induction under monocell culture conditions (DMEM medium containing 10% FBS and 1 g/mL glucose (the base medium)) (Figure 1(a)). These changes were absent in the EGFP-ANXA3 K562^R^ cells [25, 26]. When the EGFP-ANXA3 K562 cells were examined under CLSM at several time points after the haemin induction under monocell culture conditions, the EGFP-ANXA3 was translocated to the cell surface to form membrane blebbing-like vesicles, similar to those detected in apoptotic cells, and on day 8 after the haemin induction, the cell was decreased in size (Figure 1(b)). Conversely, these morphological changes were initiated in the EGFP-ANXA3 K562^R^ cells for 8 days after the haemin induction, indicating that programmed cell death plays a role in promoting the haemin-induced differentiation of the K562 cells. When the K562 cells were cultured for 20 days under basic cell culture conditions, almost all the K562 cells exhibited the morphological features of necrosis (data not shown).

### 3.2. Roles of the C5aR in the Haemin-Induced Differentiation of K562 Cells under Monocell Culture Conditions

**Figure 2:**
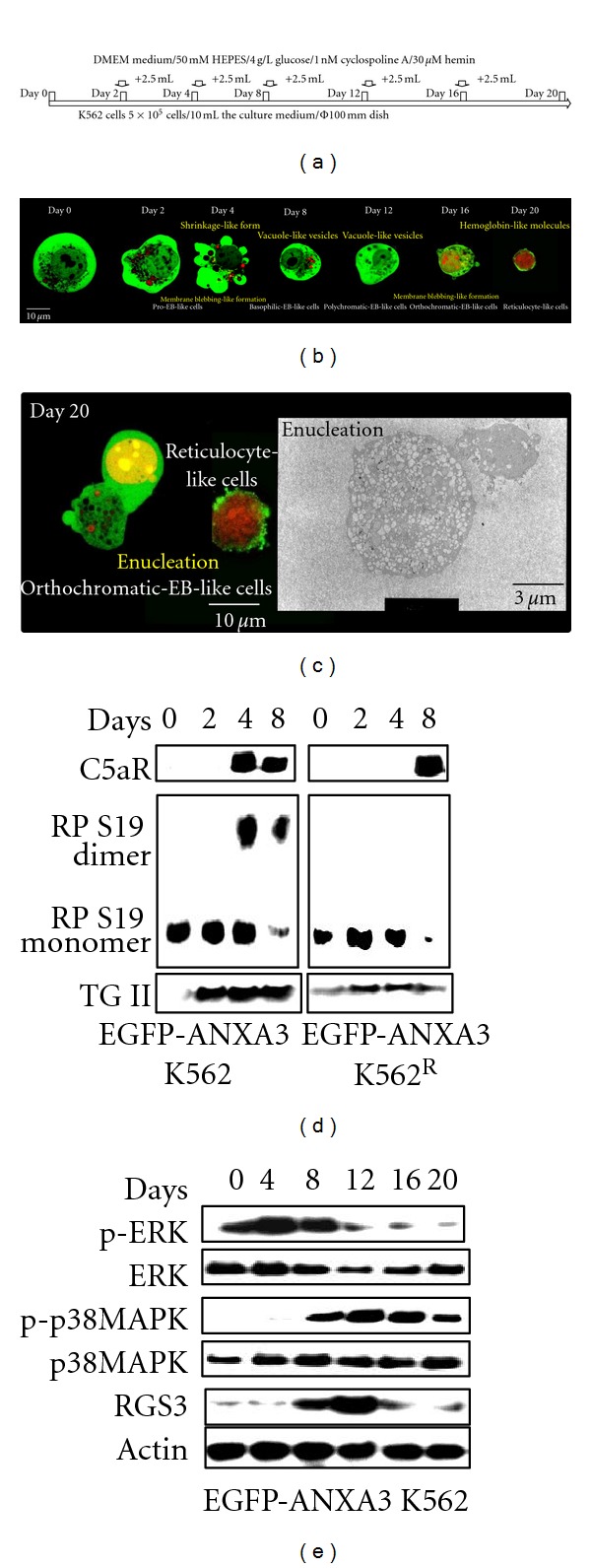
Contribution of the C5aR-RP S19 oligomer system to the haemin-induced differentiation of K562 cells under monocell culture conditions (a) Time schedule for the K562 monocell culture. ((b) and (c)) K562 cells expressing EGFP-ANXA3 were observed under CLSM or TEM at several time points after the haemin induction (*n* = 6). ((d) and (e)) EGFP-ANXA3-expressing K562 and K562^R^ cells were harvested at several time points after the haemin induction. After transferring the proteins from the SDS gel onto the membrane, the first immunoblotting was performed using the rabbit IgGs against C5aR, RP S19, and TGII (d) or ERK1/2, p38MAPK, RGS3, and actin (e) (*n* = 3).

To overcome the haemin-induced cell differentiation defect of the K562 cells, the composition of the culture medium was modified by adding 50 mM HEPES, 3 g/L glucose and 1 nM cyclosporine A to the base culture medium to stabilise the pH and to promote the glycolysis and calcineurin-dependent homeostasis of the cells [27] (Figure 2(a)).

Using CLSM, the morphological changes in the membrane blebbing-like formations were observed on days 4 and 16, the vacuole-like vesicles [28, 29] on days 12 and 16, and the haemoglobulin-like molecules on day 20 after the haemin induction in the differentiated EGFP-ANXA3 K562 cells (Figure 2(b)). In addition, we occasionally observed a large number of the vacuole-like vesicles using CLSM and transmission electron microscopy (TEM, Hitachi H-300, Hitachi, Tokyo, Japan) on day 12 after haemin induction, and nuclei in the vacuole-like vesicles were observed 16 days after the haemin induction (see Figures  1(a) and  1(b) in Supplemental Material available at doi:10.1155/2012/187080). Moreover, an increase in the LC3-II expression in the differentiated EGFP-ANXA3 K562 cells was detected by western blotting (see Figure  1(c) in Supplemental Material). These data indicated that the cells were differentiated into EGFP-ANXA3 K562 polychromatic-EB-like cells at 12 days after the haemin induction under the monocell culture conditions. 

Typically, the EGFP-ANXA3 was translocated to the perinuclear in the differentiated EGFP-ANXA3 K562 cells during the 20 days after haemin induction, indicating that the K562 orthochromatic-EB-like cells were differentiated into reticulocyte-like cells and pyrenocyte-like cells. In the reticulocyte-like cells, the EGFP-ANXA3 had disappeared and haemoglobin-like molecules were generated, and in the pyrenocyte-like cells, the membrane blebbing-like formations were induced to allow for phagocytic clearance (Figure 2(c)) [30]. These morphological findings indicated a transition into the EGFP-ANXA3 K562 orthochromatic EB-like cell stage by day 18 after the haemin induction under monocell culture conditions. However, 99.9% of the differentiated EGFP-ANXA3 K562 cells underwent necrosis even under the modified base culture conditions. Only 0.1% of the EGFP-ANXA3 K562 erythrocyte-like cells (data not shown) were recovered. 

The morphological changes in the K562 cells after the MnCl_2_ treatment were similar to the changes associated with the haemin induction (Figure 1), indicating that NF-Y binds to *C5aR* and that RP S19 oligomers were generated because of cross-linking by the activated TGII. To confirm the activities of NF-Y and RP S19, the differentiated EGFP-ANXA3 K562 cells were harvested at several time points after the haemin induction and were analysed by western blotting. The expression of the C5aR, RP S19 dimer, and TGII was increased on day 8 after the haemin induction (Figure 2(d)); the expression of the proteins was delayed in the EGFP-ANXA3 K562^R^ cells. 

We previously reported that RGS3 promotes pro-apoptosis by downregulating ERK1/2 phosphorylation [23]. In addition to the C5aR, we recently discovered the expression of RGS3 on apoptotic cells. MAPK phosphorylation was switched from the ERK1/2 pathway to the p38MAPK pathway in the EGFP-ANXA3 K562 prochromatic-EB-like cells following the expression of RGS3 (Figure 2(e)). These data indicated that the *de novo* synthesised C5aRs that are bound to the RP S19 dimer are released in the K562 basophilic-EB-like cells to promote haemin-induced erythropoiesis byswitching the MAPK signalling from the ERK1/2 pathway to the p38MAPK pathway through the expression of RGS3.

### 3.3. Roles of RP S19 Oligomers in the Haemin-Induced Differentiation of K562 Cells under Monocell Culture Conditions

**Figure 3:**
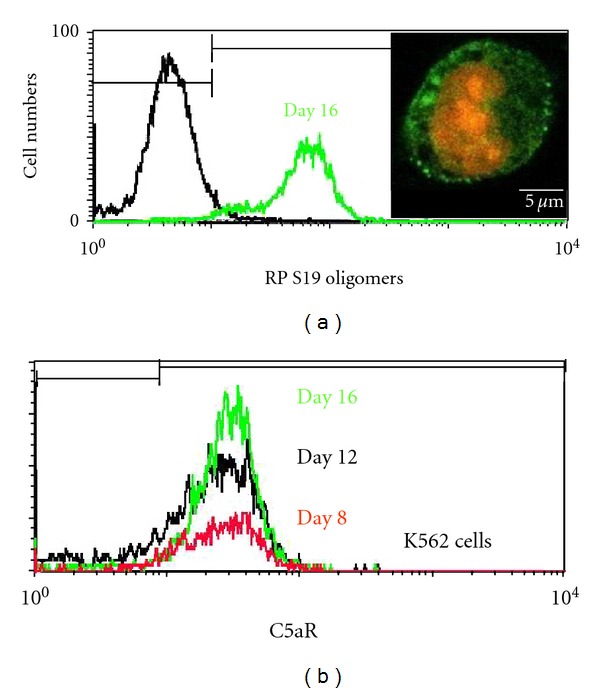
Generation of RP S19 oligomers and expression of the C5aR during the haemin-induced differentiation of K562 cells under monocell culture conditions K562 cells were harvested at several time points after the haemin induction and were stained with anti-C5a rabbit IgG or anti-C5aR mouse IgG for the FACS and CLSM analyses (*n* = 3).

Recently, we demonstrated that the RP S19 oligomers are distinguishable from the monomers by immunoblotting with anti-human C5a rabbit IgG [20]. Under our current experimental conditions, the monocyte chemotactic activity of the K562 basophilic-EB-like cell supernatant was neutralised not only by anti-RP S19 rabbit IgG but also by anti-C5a rabbit IgG (data not shown). When the supernatant of the K562 basophilic-EB-like cells was analysed using SDS-PAGE and western blotting with anti-RP S19 rabbit IgG and anti-C5a rabbit IgG, a 34-kDa protein band was shown to cross-react with both rabbit IgGs (data not shown). The RP S19 oligomers in the cytoplasmic and extraplasmic plasma membranes of the K562 orthochromatic EB-like cells were detected under CLSM and by FACS using anti-C5a rabbit IgG (Figure 3). Conversely, using FACS, the C5aR was detected on the extraplasmic plasma membranes of the K562 cells from the basophilic-EB-like cell stage to the orthochromatic-EB-like cell stage because the C-terminus of the C5aR was not phosphorylated by downstream signals when the C5aR was bound to RP S19 oligomers, as previously shown in HL-60 cells [6].

**Figure 4:**
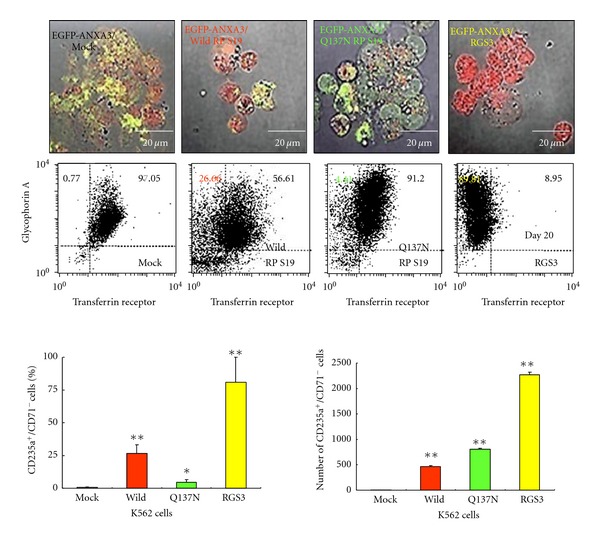
Contribution of the C5aR-RP S19 oligomer system to the haemin-induced terminal differentiation of K562 cells under monocell culture conditions. K562 cells expressing EGFP-ANXA3/Mock, EGFP-ANXA3/Wild-type RP S19, EGFP-ANXA3/Q137N mutant RP S19, and EGFP-ANXA3/RGS3 were harvested at several time points after the haemin induction and analysed under CLSM (*n* = 3). The mock (black), Wild-type RP S19 (red), Q137N mutant RP S19 (green), and RGS3 (yellow) K562 cells were harvested at several time points after the haemin induction and stained with FITC-conjugated anti-CD71 and APC-conjugated anti-CD235a mouse IgGs for FACS analyses (*n* = 3). The averages of the percentage and number of CD235a^+^/CD71^−^ erythrocyte-like cells were calculated on day 20 of the monocell culturing. *P* values of less than 0.05 were considered statistically significant (*P < 0.05 or **P < 0.01).

Next, to investigate the dominant negative effects of the nonfunctional Q137N RP S19 oligomers on the haemin-induced differentiation of K562 cells by the neutrophil-like C5aR without receptor internalisation, we transfected the EGFP-ANXA3 K562 cells or the nonlabelled K562 cells with the Wild-type RP S19 and Gln137Asn mutant RP S19 cDNAs (Mock, Wild RP S19 and Q137N RP S19 K562 cells). As shown in Figure 4, almost all the EGFP-ANXA3/Mock K562 erythrocyte-like cells were necrotic and did not demonstrate clear plasma membranes when grown under monocell culture conditions on day 20 after the haemin induction. 

To confirm these pathological findings, the Mock K562 erythrocyte-like cells not expressing EGFP-ANXA3 were collected for the FACS analysis of CD235a and/or CD71 expression (Figure 4). Using FACS, we detected 0.77 ± 0.12% of CD235a^+^/CD71^−^ cells (5.9 cells) in 0.1 ± 0.2% of the cells exhibiting viability rates of 15.3 ± 6.4% (7.7 × 10^2^ cells) recovered from the starting Mock K562 cells (5 × 10^7^ cells), indicating that the necrotic cells resembled the haemin-induced K562 erythrocyte-like cells.

In contrast to the EGFP-ANXA3/Mock K562 cells, the EGFP-ANXA3/Wild RP S19 K562 erythrocyte-like cells occasionally exhibited red spots on their clear plasma membranes that were caused by the production of haemoglobin-like molecules. Conversely, the differentiated EGFP-ANXA3/Q137N RP S19 K562 cells exhibited green spots on their clear plasma membranes, indicating that haemoglobin-like molecules were not produced. Using FACS, we detected 26.66 ± 6.43% of CD235a^+^/CD71^−^ cells (4.7 × 10^2^ cells) in 1.0 ± 0.4% of the cells exhibiting viability rates of 35.1 ± 14.7% (1.8 × 10^3^ cells) recovered from the starting Wild RP S19 K562 cells. In contrast, we detected 4.41 ± 2.14% of CD235a^+^/CD71^−^ cells (8.0 × 10^2^ cells) in 8.0 ± 4.1% of the cells exhibiting viability rates of 45.6 ± 4.7% (1.8 × 10^4^ cells) recovered from the starting Q137N RP S19 K562 cells (5 × 10^7^ cells). These data indicated that the effect of the RP S19 oligomers on the pro-apoptotic signal is crucial for the haemin-induced K562 cell differentiation.

To examine the direct effects of RGS3, induced by the RP S19 oligomer-C5aR binding, on the haemin-induced differentiation of K562 cells, we transfected the EGFP-ANXA3 K562 cells or non-labelled K562 cells with the RGS3 cDNA (RGS3 K562 cells). The EGFP-ANXA3/RGS3 K562 erythrocyte-like cells with clear plasma membranes exhibited red spots caused by the production of haemoglobin-like molecules (Figure 2(b)). Using FACS, we routinely detected 80.81 ± 19.28% of CD235a^+^/CD71^−^ cells (2.3 × 10^4^ cells) in 1.0 ± 0.4% of the cells exhibiting viability rates of 56.2 ± 12.1% (2.8 × 10^2^ cells) recovered from the starting RGS3 K562 cells.

These data indicated that the apoptotic C5aR was expressed and the RP S19 oligomers were generated in the K562 EB-like cells. The C5aR bound by RP S19 oligomers, which were released from the K562 basophilic-EB-like cells in an autocrine manner, induced the initiation of the transcription of *RGS3* without receptor internalisation. The RGS3 decreased the ERK1/2 signalling and shifted the pro-apoptotic signal towards apoptosis. We only showed the modulation of one signalling pathway during the haemin-induced differentiation of the K562 cells.

### 3.4. Effects of Coculturing with THP-1 Macrophage-Like Cells on the Haemin-Induced Differentiation of K562 Cells

**Figure 5:**
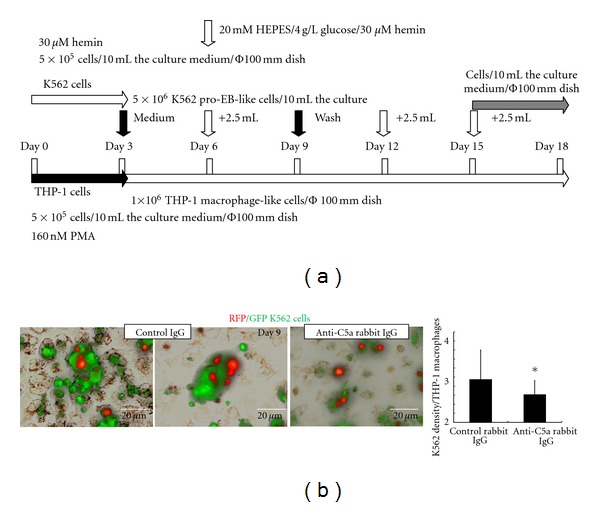
Contribution of the RP S19 oligomers to the K562-THP-1 cell interaction (a) Time schedule for the K562 cell that were cocultured with the THP-1 cells. (b) K562 cells expressing RFP-Nucleus/EGFP-ANXA3 were cultured for 9 days after the haemin induction and were examined under fluorescence microscopy. The microscopic images (4 pairs) were used to count the number of K562 cells on each THP-1 macrophage-like cell using the Image-J software (*n* = 3). *P* values of less than 0.05 were considered statistically significant (*P < 0.05).

To examine the effects of coculturing with macrophages on the haemin-induced differentiation of K562 cells, we used K562 cells expressing the RFP nuclear entry signal-Tag and EGFP-ANXA3 cDNAs (RFP/EGFP K562 cells) and unlabelled THP-1 cells. The RFP/EGFP K562 cells differentiated in the culture medium within 3 days after the haemin induction. The differentiated RFP/EGFP K562 cells were washed 3 times with PBS, and 5 × 10^6^ cells were resuspended in 10 mL of the culture medium containing the control rabbit IgG or the anti-C5a rabbit IgG. The cells were mixed with the THP-1 macrophage-like cells (5 × 10^5^ cells) that were treated with PMA for 3 days (Figure 5(a)). The K562 cells that were not bound to the THP-1 macrophage-like cells were removed using 3 PBS washes on day 6 of the coculturing, and the remaining RFP/EGFP K562 basophilic EB-like cells that were bound to the THP-1 macrophage-like cells were examined under fluorescent microscopy (IX70, Olympus) (Figure 5(b)). The fluorescent images were analysed using the NIH Image-J software to determine the number of bound K562 basophilic EB-like cells, which were visible as solid red circles. In the presence of the control rabbit IgG, 3.2 ± 0.35 RFP/EGFP K562 basophilic EB-like cells were bound to one THP-1 macrophage-like cell. However, the number of bound RFP/EGFP K562 basophilic EB-like cells to each THP-1 macrophage-like cell was reduced significantly to 2.6 ± 0.32 in the presence of the anti-C5a rabbit IgG. 

To examine the effects of coculturing on further differentiation of the cells, the K562 cells that were not bound to the THP-1 macrophage-like cells were harvested from the culture medium on days 9 and 15 after the haemin induction and were analysed using FACS and CLSM. Using FACS, the K562 orthochromatic-EB-like cells (R2 in Figure 2(a) in Supplemental Material) and the relatively small K562 reticulocyte-like cells without nuclei (R3 in Figure 2(a) in Supplemental Material) were clearly distinguishable as RFP^high^/GFP^high^ cells and RFP^middle^/GFP^middle^ cells, respectively. In addition, we prepared K562 cells or THP-1 cells bearing the RFP cDNA or the EGFP-ANXA3 cDNA, and the RFP K562 pyrenocyte-like cells were observed in the EGFP-ANXA3 THP-1 macrophage-like cells under CLSM on day 15 after the haemin induction (see Figure 2(b) in Supplemental Material).

The EGFP-ANXA3 K562 reticulocyte-like cells that were disassociated from the unlabelled THP-1 macrophage-like cells were harvested from the culture medium on day 15 after the haemin induction and were recultured in the culture medium in the absence of haemin for additional 3 days. Using fluorescence microscopy (IX70), the EGFP-ANXA3 K562 basophilic-like cells containing the RFP-labelled nuclei were shown to colocalise with the unlabelled THP-1 macrophage-like cells (see Figure 3(a) in Supplemental Material). Only the cells containing the RFP-labelled nuclei colocalised with the unlabelled THP-1 macrophage-like cells (see Figure 3(b) in Supplemental Material). Conversely, we detected the EGFP-ANXA3 K562 reticulocyte-like cells with no RFP-labelled nuclei in the culture supernatants using CLSM (see Figure 3(c) in Supplemental Material). Interestingly, the EGFP-ANXA3 disappeared in the K562 reticulocyte-like cells and induced the production of haemoglobin-like molecules when the cells were cultured for additional 3 days in the absence of haemin (see Figure 3(d) in Supplemental Material).

### 3.5. Direct Observation of the Dominant Negative Effect of Gln137Asn Mutant RP S19 Overexpression on the Interaction of K562 Basophilic-EB-Like Cells with the THP-1 Macrophage-Like Cells Using Time-Lapse Microscopy

**Figure 6:**
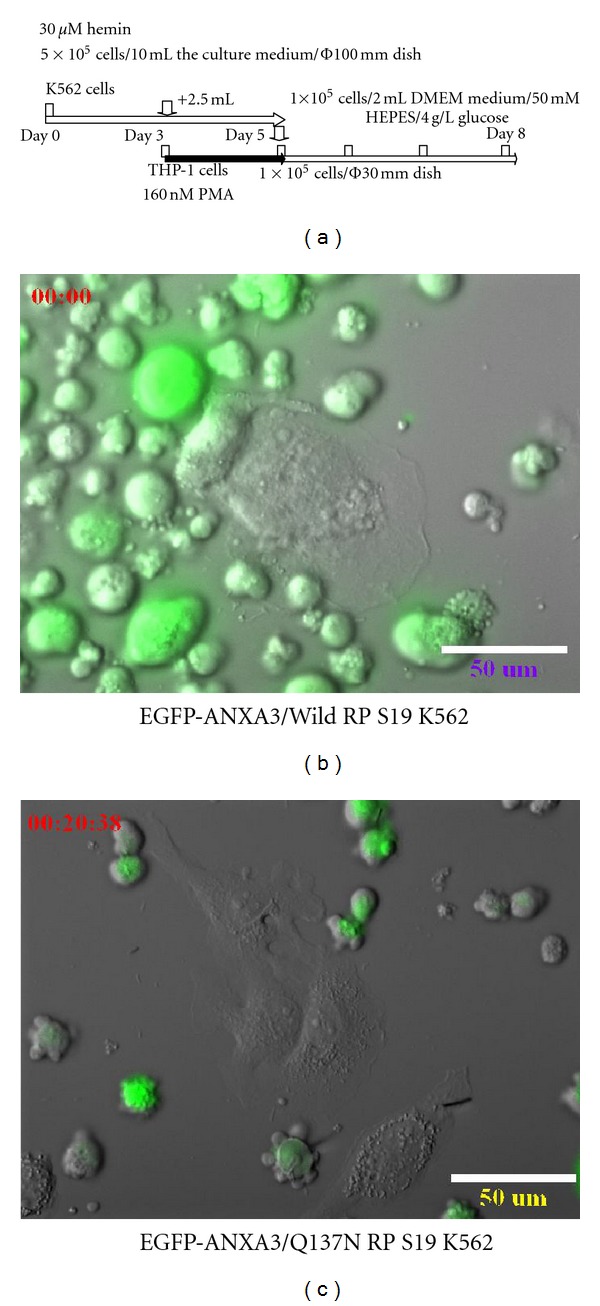
Detection of the contribution of the K562-THP-1 cell interaction to the haemin-induced terminal differentiation of K562 cells using time-lapse microscopy. (a) Time schedule for the K562 cells that were cocultured with the THP-1 cells. K562 cells expressing EGFP-ANXA3/Wild-type RP S19 (b) and EGFP-ANXA3/Gln137Asn mutant RP S19 (c) were cultured with the THP-1 macrophage-like cells for 1–3 days and monitored using the LCV110 microscope (*n* = 3).

To confirm the data using time-lapse microscopy, we used the EGFP-ANXA3/Wild RP S19 K562 cells, the EGFP-ANXA3/Q137N RP S19 K562 cells, and the unlabelled THP-1 cells. The K562 cells that differentiated in the culture medium within 5 days of the haemin induction were washed 3 times with 3 mL of PBS, and 1 × 10^5^ cells were resuspended in 2 mL of fresh culture medium in the absence of haemin (Figure 6(a)). In contrast to the K562 cells, the THP-1 cells were differentiated into macrophage-like cells within 2 days of PMA treatment in a glass-bottom dish. The cells were cocultured and were examined under the fluorescence microscope LCV110 (Olympas) for 1–3 days.

The EGFP-ANXA3/Wild RP S19 K562 basophilic-EB-like cells bound to the THP-1 macrophage-like cells within 12 hr of coculturing and started to move about on the THP-1 macrophage-like cells (Figure 6(b)). The haemoglobin-like molecules appeared in a time-dependent manner. However, the EGFP-ANXA3/Q137N RP S19 K562 pro-EB-like cells did not bind to the THP-1 macrophage-like cells during the 48 hr coculturing (Figure 6(c)). 

### 3.6. Direct Observation of Effects of RP S19 Oligomer Inhibitor and the C5aR Inhibitor on the Interaction of the K562 Basophilic-EB-Like Cells with the THP-1 Macrophage-Like Cells Using Time-Lapse Microscopy

**Figure 7:**
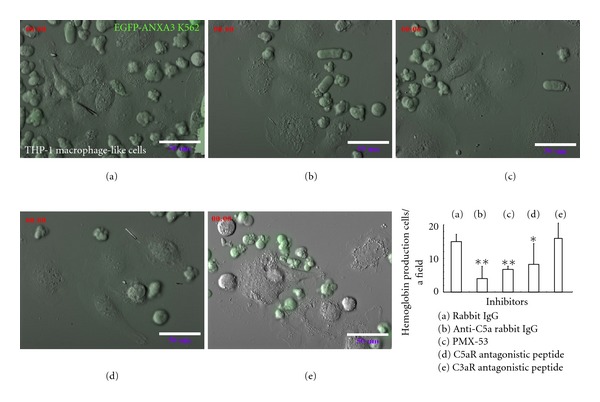
Detection of the inhibition of the K562-THP-1 cell interactions by the inhibitors of the C5aR-RP S19 oligomer system using time-lapse microscopy. ((a)–(e)) EGFP-ANXA3-overexpressing K562 cells were cultured with the THP-1 macrophage-like cells in the presence of control rabbit IgG, anti-C5a rabbit IgG, PMX-53, W-54011, or SB 290157 for 1–3 days and were recorded using the LCV110 microscope (*n* = 3). The maximal number of cells containing haemoglobin-like molecules at 72 hr of coculturing was counted automatically from 4 random fields, and the averages were calculated. *P* values of less than 0.05 were considered statistically significant (*P < 0.05 or **P < 0.01).

The haemin-induced differentiation of the EGFP-ANXA3 K562 cells on the THP-1 macrophage-like cells within 72 hours of coculturing was reproducible. This cellular differentiation was not modified by the presence of either the control rabbit IgG (5 *μ*g/mL) or the C3aR antagonistic peptide (10^−6^ M SB 290157) (Figures 7(a) and 7(e)). However, the interaction between the K562 basophilic-EB-like cells and the THP-1 macrophage-like cells was inhibited when the RP S19 oligomers were neutralised using anti-C5a rabbit IgG (5 *μ*g/mL) or when the ligand-binding site of the C5aR was blocked using the C5aR antagonistic peptides (10^−6 ^M PMX-53 and W-54011) (Figures 7(b)–7(d)), preventing the haemin-induced differentiation of the K562 cells. The average maximal number of cells containing haemoglobin-like molecules from 4 random fields at 72 hr after coculturing was 15.00 ± 2.16 and 15.25 ± 2.06 in the presence of the control rabbit IgG and SB 290157, respectively. However, the number of red cells decreased to 4.00 ± 3.65, 6.75 ± 0.96, and 8.25 ± 6.13 in the presence of the anti-C5a rabbit IgG, PMX-53, and W-54011, respectively. 

## 4. Discussion

### 4.1. The RP S19 Oligomer-C5aR System in Apoptotic Cells

We have demonstrated that the RP S19 monomer translocates to the cytoplasmic plasma membrane in almost all apoptotic cells and interacts with the Lys_33_–Lys_39_ moiety of the negatively charged phosphatidylserine [31, 32]. After the cross-linking of residues Lys122 and Gln137 by an activated TGII, RP S19 oligomers are released by the flip/flop system and function as monocyte C5aR ligands to induce migrating cells into apoptosis [33]. Interestingly, we have previously reported that the NF-Y transcription factor binds to the CCAAT box in the promoter region of *C5aR* even though the cells do not constantly express *C5aR* [23]. When the RP S19 oligomers bind to the apoptotic C5aR, *RGS3* is expressed and the programmed cell death process is induced with the decrease in the ERK1/2 signalling. Phosphatidylserine exposure on the extraplasmic plasma membrane is crucial for recognition by macrophages [34]. In addition, microparticles, such as ectosomes and exosomes, bud directly from the cell surface of apoptotic cells accompanied by the loss of cell organelles [35, 36]. The RP S19 oligomer-C5aR system appears to synchronise the execution of apoptosis with the recruitment of macrophages. This mechanism may explain the maintenance of homeostasis without inflammatory cues.

### 4.2. C5aR on the Haemin-Induced Differentiation of K562 Cells

The *C5aR* is expressed not only in myeloid cells but also in various nonmyeloid cells including microvascular endothelial cells, articular chondrocytes, and stimulated hepatocytes [3]. Interesting studies have reported that the erythroid-specific transcriptional activation of *growth factor-independence-1B* and *nucleolar spindle-associated protein* genes contributes to the functional cooperation between GATA-1 and NF-Y in immature K562 cells and mature erythroleukemia MEL cells [16, 17]. This study may be the first report the generation of the C5aR protein on the cell surface of K562 cells during the pro-EB-like cell stage to the orthochromatic EB-like cell stage (Figure 3).

### 4.3. The RP S19 Oligomer-C5aR System during the Haemin-Induced Differentiation of K562 Cells

In this study, we generated the EGFP-ANXA3 K562 cells to examine the RP S19 oligomer-C5aR system using microscopy. ANXA3 binds to the phosphatidylserine of dying cells using its calcium-binding and lipid-interacting moieties as markers for macrophages [19]. Interestingly, we observed membrane blebbing-like formations composed of vacuole-like vesicles that contained high amounts of EGFP-ANXA3 in the K562 pro-EB-like cells, similar to those detected in apoptotic K562 cells after 6 hr of MnCl_2_ treatment (Figure 1). Similar morphological features were reported during erythropoiesis to allow the clearance of the transferrin receptor and the loss of cell organelles in apoptotic cells [36]. We propose that the RP S19 monomer in the K562 EB-like cells moves to the phosphatidylserine on the cytoplasmic plasma membrane and is cross-linked by TGII, similar to the events that occur during apoptosis [4]. Interestingly, we detected fluorescence spots that were relatively large in size and cross-reacted with anti-C5a rabbit IgG not only on the cytoplasmic plasma membrane but also in the cytoplasm (inside in Figure 3). Recently, vesicle curvature has been shown to change the optimal binding of lactadherin to phosphatidylserine in apoptotic cells [37]. These data suggested that the RP S19 monomer in the K562 EB-like cells translocates to the phosphatidylserine on the cytoplasmic plasma membrane and on vacuole-like vesicles.

### 4.4. Effects of the Interactions of the THP-1 Macrophage-Like Cells with the K562 Basophilic-EB-Like Cells during Erythropoiesis

Bone marrow macrophages play an important role in creating a special microenvironment for EBs [38]. For example, the interaction between the erythroblast intercellular adhesion molecule-4 with the macrophage *α*V integrin is crucial for the formation of the EB-macrophage island [39]. In this study, we demonstrated that the interaction of the K562 basophilic-EB-like cells with the THP-1 macrophage-like cells was inhibited by overexpressing the Gln137Asn mutant RP S19, by neutralising the RP S19 oligomers using anti-human C5a rabbit IgG or by blocking C5aR with the C5aR antagonistic peptides (Figures 5–7). Our data indicated that the K562 basophilic-EB-like cells express the C5aR and generate RP S19 oligomers. The released RP S19 oligomers attract the THP-1 macrophage-like cells, and both cells are connected by RP S19 oligomers via their C5aRs without the internalisation of the receptor. The K562 basophilic EB-like cells move about on the THP-1 macrophage-like cells through the interaction of erythroblast-associated molecules with the macrophage integrins. However, there are no reports of erythroblastopenia in *C5aR* knockout mice; it is likely that redundant mechanisms involving other chemotactic receptors exist in these knockout mice [40].

The morphological changes associated with the maturation of haemin-induced K562 cells *in vitro* under monocell culture conditions take four times longer than the process of erythroid maturation in the bone marrow (Figure 2(b)); however, the maturation process was shortened by the *in vitro* coculture conditions used (Figures 5(a) and 6(a)). Only 5.9 K562 erythrocyte-like cells were recovered from the 5 × 10^7^ K562 cells under monocell culture conditions (Figure 4); however, a relatively larger number of K562 erythrocyte-like cells were recovered following coculture with the THP-1 macrophage-like cells. These data highlight the roles of the macrophages during the haemin-induced differentiation of K562 cells. The macrophages of the EB-macrophage islands in the bone marrow negatively and positively modulate the balance between the survival and apoptotic signals of EB by releasing various cytokines, including interleukin-1, granulocyte-macrophage colony-stimulating factor, transforming growth factor-*β*, and activin, to maintain and promote erythropoiesis [39].

In contrast, the RP S19 oligomers released from the K562 basophilic-EB-like cells activated both the K562 erythrocyte-like cells and the THP-1 macrophage-like cells via the C5aR. However, when the K562 basophilic-EB-like cells and the THP-1 macrophage-like cells were cultured in trans-well chambers, we observed low numbers of the K562 erythrocyte-like cells (see Figure 4 in Supplemental Material). Interestingly, we occasionally observed the EGFP-ANXA3 from the K562 cells as green spots in the THP-1 macrophage-like cells (see Figure 3(b) in Supplemental Material). Therefore, we need to examine further the direct transfer of intracellular proteins between the basophilic-EB-like cells and the bone marrow macrophages.

## Supplementary Material

Supplementary Figure1: Morphological changes of EGFP-ANXA3 K562 cells during the hemin-induced differentiation under monocell culture conditions
Supplementary Figure2: Effects of the K562-THP-1 cell interaction on the hemin-induced erythropoiesis
Supplementary Figure3: x1000x1000x40x40Effects of the K562-THP-1 cell interaction on the hemin-induced terminal differentiation
Supplementary Figure4: Roles of macrophages in the hemin-induced cell differentiation of K562 cells
