# Supplementary material for: Dual Functions of the C5a Receptor as a Connector for the K562 Erythroblast-Like Cell-THP-1 Macrophage-Like Cell Island and as a Sensor for the Differentiation of the K562 Erythroblast-Like Cell during Haemin-Induced Erythropoiesis
Source: Clin Dev Immunol. 2012 Dec 30;2012:187080. doi: 10.1155/2012/187080 (PMC3546471; doi:10.1155/2012/187080)
Supplement: Supplementary file 1 — Supplementary Figure1: Morphological changes of EGFP-ANXA3 K562 cells during the hemin-induced differentiation under monocell culture conditions Supplementary Figure2: Effects of the K562-THP-1 cell interaction on the hemin-induced erythropoiesis Supplementary Figure3: x1000x1000x40x40Effects of the K562-THP-1 cell interaction on the hemin-induced terminal differentiation Supplementary Figure4: Roles of macrophages in the hemin-induced cell differentiation of K562 cells [file 187080.f1.pdf]

Supplemental Figure 1

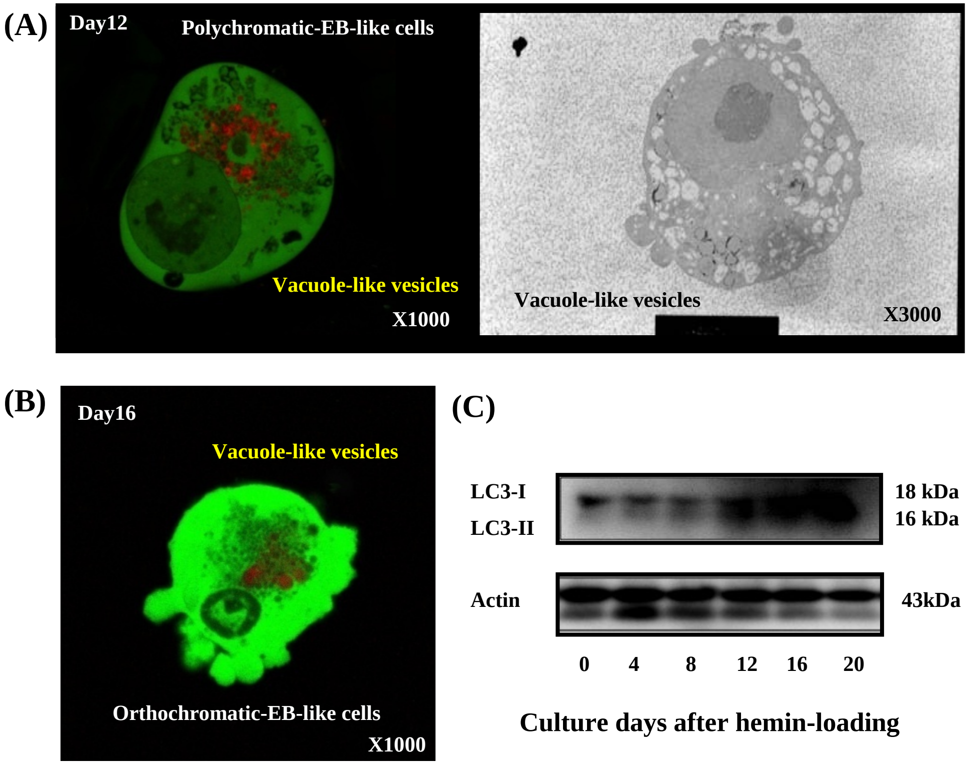

Morphological changes of EGFP-ANXA3 K562 cells during the hemin-induced differentiation under monocell culture conditions (A and B) K562 cells expressing EGFP-ANXA3 were observed by CLSM or TEM at 12 and 16 days after hemin induction (n=6). (C) EGFP-ANXA3-expressing K562 cells were harvested at several time points after hemin induction. After transferring the proteins from the SDS gel onto the membrane, the first immunoblotting was performed with the rabbit IgGs against LC3 and actin (n=3).

Supplemental Figure 2

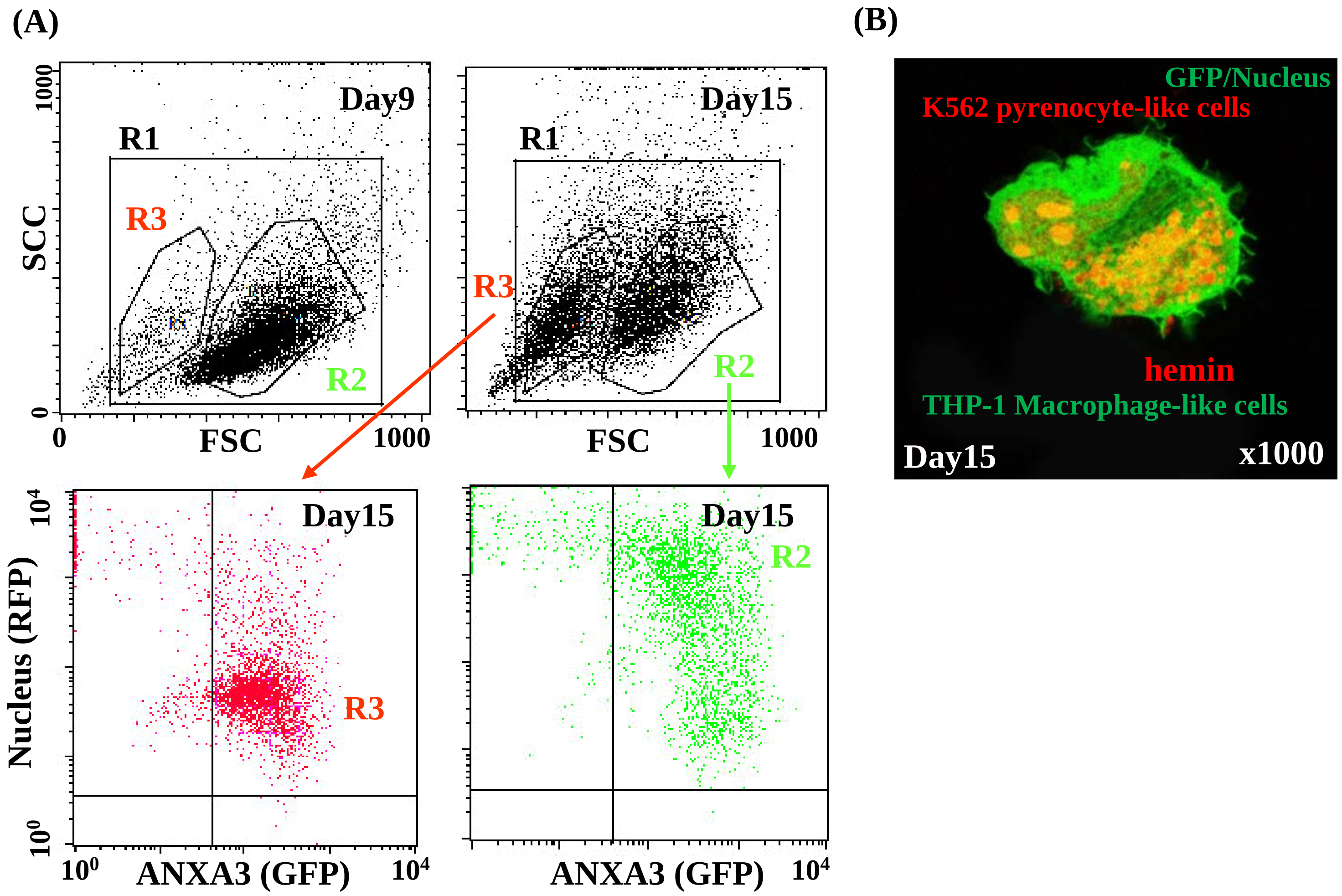

Effects of the K562-THP-1 cell interaction on the hemin-induced erythropoiesis (A) K562 cells expressing RFP-Nucleus/EGFP-ANXA3 were harvested at several time points after hemin induction and analysed by FACS (n=3). (B) After co-culturing of the EGFP-ANXA3 THP-1 macrophage-like cells with the RFP-Nucleus K562 cells on a chamber slide, the THP-1 macrophage-like cells at day 15 after hemin induction were observed by CLSM (n=3).

Supplemental Figure 3

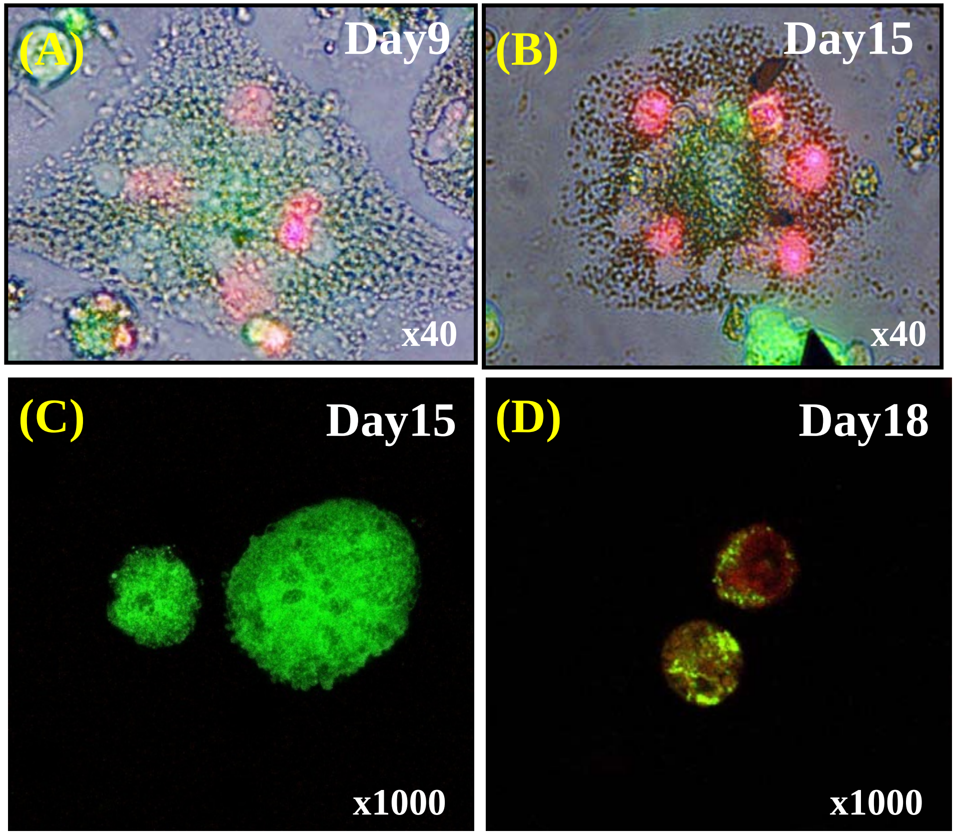

Effects of the K562-THP-1 cell interaction on the hemin-induced terminal differentiation (A-D) After co-culturing of the RFP-Nucleus/EGFP-ANXA3-expressing K562 cells with the unlabelled THP-1 macrophage-like cells, the unbound and associated K562 cells at day 9 and day 15 after hemin induction were re-cultured and monitored by fluorescence microscopy and CLSM (n=3).

Supplemental Figure 4

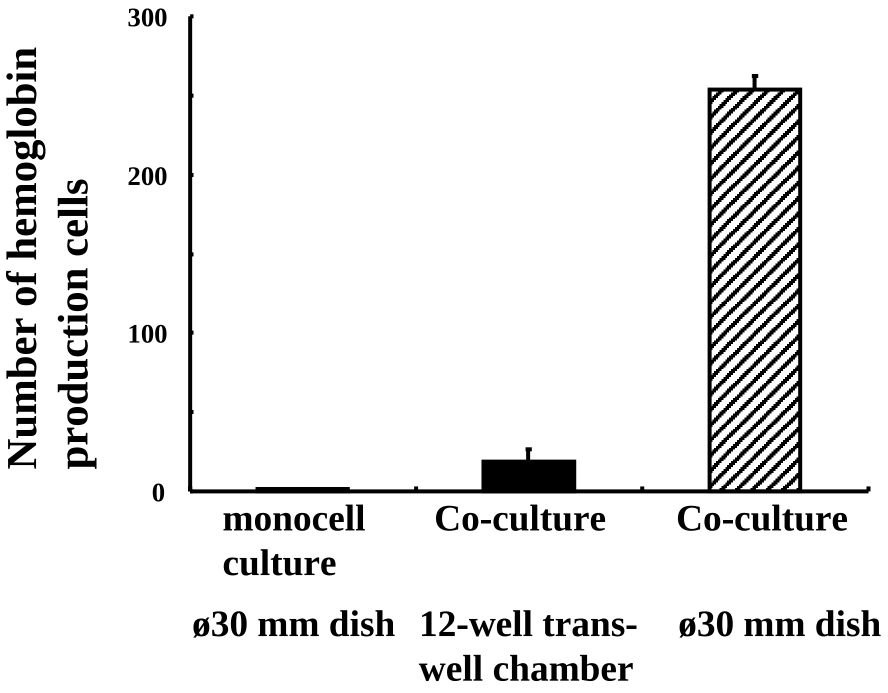

Roles of macrophages in the hemin-induced cell differentiation of K562 cells
1 x 10^5 Mock K562 cells were cultured in ø30 mm dish under either the monocell culture condition or the co-culture condition and in 12-trans-well chamber under the co-culture condition. The averages of number of hemoglobin production cells were counted by FACS. (n=4).
